# Unravelling immune microenvironment features underlying tumor progression in the single-cell era

**DOI:** 10.1186/s12935-024-03335-z

**Published:** 2024-04-22

**Authors:** Qilian Du, Qi An, Jiajun Zhang, Chao Liu, Qinyong Hu

**Affiliations:** 1 Department of Oncology, Renmin Hospital of Wuhan University, Wuhan, 430060 China; 2 Department of Radiation Oncology, Peking University First Hospital, Beijing, 100034 China

**Keywords:** Single-cell RNA sequencing, Immune microenvironment, Tumorigenesis, Tumor development

## Abstract

The relationship between the immune cell and tumor occurrence and progression remains unclear. Profiling alterations in the tumor immune microenvironment (TIME) at high resolution is crucial to identify factors influencing cancer progression and enhance the effectiveness of immunotherapy. However, traditional sequencing methods, including bulk RNA sequencing, exhibit varying degrees of masking the cellular heterogeneity and immunophenotypic changes observed in early and late-stage tumors. Single-cell RNA sequencing (scRNA-seq) has provided significant and precise TIME landscapes. Consequently, this review has highlighted TIME cellular and molecular changes in tumorigenesis and progression elucidated through recent scRNA-seq studies. Specifically, we have summarized the cellular heterogeneity of TIME at different stages, including early, late, and metastatic stages. Moreover, we have outlined the related variations that may promote tumor occurrence and metastasis in the single-cell era. The widespread applications of scRNA-seq in TIME will comprehensively redefine the understanding of tumor biology and furnish more effective immunotherapy strategies.

## Introduction

The occurrence and development of tumors is a multi-step complex process caused by intra- and extra-tumoral factors, accompanied by profound dynamic variations in the tumor microenvironment (TME) [1]. Tumor immune microenvironment (TIME) refers to the immune cell populations and molecules around tumor cells in TME [2]. The immune system can partially destroy malignant cells. However, its effectiveness declines due to various factors, tumor neoantigens immune editing, immunosuppressive cell recruitment, and immune effector cell depletion [3, 4]. Moreover, the cell clusters and molecules of TIME vary with tumor evolution [5]. Tumors gradually evade immune surveillance and achieve expansion and metastasis through the subtle crosstalk of tumor cells and TIME [6]. Most researchers have focused on the biological characteristics of T-cells and their immune checkpoints. Therefore, significant progress has been achieved in developing clinically effective immunotherapies for multiple neoplasms, especially immune checkpoint inhibitors [5, 7–9]. However, the efficacy of these immunotherapies is limited to a minority of patients, given that tumor progression is influenced by various immune cells and their intercellular communication, not merely T-cells. Therefore, identifying altered immune-cell clusters and molecules in TIME is highly relevant in developing new immunotherapies to inhibit tumorigenesis and progression for cancer patients.

Traditional methods, including flow cytometry and bulk RNA sequencing, can provide certain features of immune cells. However, the constraints of these traditional techniques impede a thorough characterization of TIME, the phenotype and function of immune cells [10, 11]. The homogeneity of these traditional methods masks the heterogeneity of different immune cells to varying degrees. The emergency of single-cell RNA sequencing (scRNA-seq) has provided a comprehensive understanding of the tumor-infiltrating immune cell alterations and their interactions with diverse cell types at various cancer stages [12–14]. ScRNA-seq has elucidated significant findings in tumorigenesis and progression research. For example, scRNA-seq has proved that different immune cells promoted tumorigenesis through various mechanisms, including T-cells [15], B-cells [16], and neutrophils [17]. During tumor metastasis, Liu et al. [18] illustrated that during tumor metastasis, SPP1^+^ tumor-associated macrophages (TAMs) promoted liver metastasis in colorectal cancer (CRC). Thus, scRNA-seq is a powerful method to discover the potential relationship between TIME dynamic alterations and tumor development, providing novel insights into cancer immunotherapy.

Therefore, this review provides a comprehensive overview of recent applications of scRNA-seq in tumor progression research. Moreover, we have discussed cellular and molecular changes in TIME, to understand the biological features of the immune system better and accurately explore novel and potent immunotherapy for cancer.

## Tumor cells reprogram TIME during cancer development

**Fig. 1:**
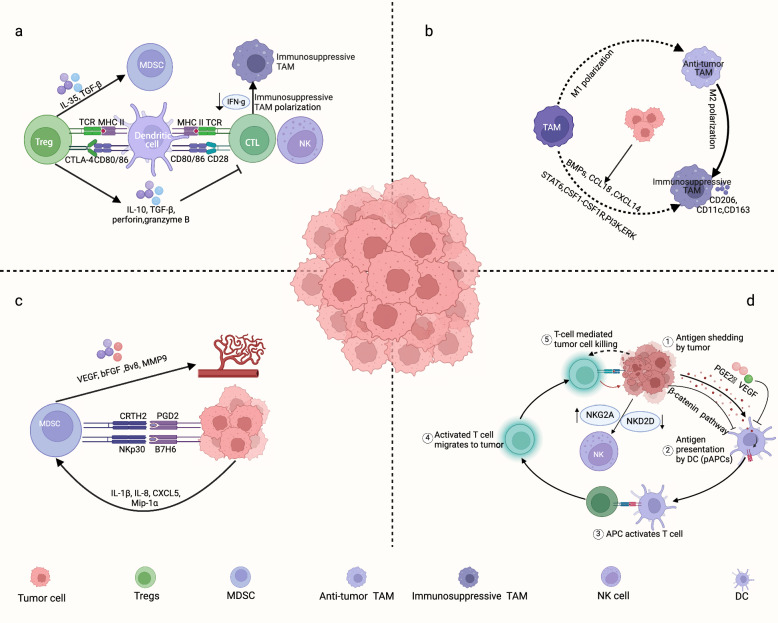
Remodeling of the immune microenvironment by tumor cells. **a** The regulation of T_reg_ influences TME through various mechanisms; **b** TAM polarization affects the tumor immune microenvironment; **c** The role of myeloid cells in tumor development; **d** The role of NK cells in the tumor immune microenvironment

The intrinsic factors of tumor cells are genetic, transcriptional, and functional alterations [19], which induce forming and maintaining an immunosuppressive TIME and hinder the ability of immune cells to identify and respond to tumor cells (Fig. 1). TAMs, regulatory T-cell (T_reg_), and tumor cells with high expression of programmed-death-ligand-1 (PD-L1) are major subsets of immunosuppressive cells in TME, which jointly participate in the immune escape and progression of tumors.

### Immunosuppressive cell recruitment and molecules expression

#### T_reg_

T_regs_ play a pivotal role in actively suppressing immune responses against the body's own tissues (self-antigens). This significantly weakens the immune system's attack on cancer cells and allows tumors to progress. Cancer cells grow rapidly and require a lot of nutrients. This creates a TME with limited nutrients, hypoxia, and a buildup of waste products (metabolites). Interestingly, T_regs_ are adaptable in their metabolism. This allows them to thrive in the TME and contribute to its immunosuppressive nature, further hindering anti-tumor immunity. Different metabolic programs control the growth, movement, and function of T_regs_ through key regulatory factors. These programs highlight the central role of T_regs_ in shaping the immune response within the TME [20]. T_reg_ produces several immune-suppressive cytokines due to cell-to-cell contact. On the one hand, T_reg_ secretes various immunosuppressive factors to inhibit CD8^+^ T-cells proliferation, including interleukin 10 (IL-10) and IL-35 [21–24]. Moreover, T_reg_ produces perforin and granzyme to kill natural killer (NK) cells and cytotoxic lymphocytes (CTL) when tumor cells are attacked [25]. On the other hand, T_reg_ cells and immunosuppressive cell clusters labored together to prevent CTL cells from secreting interferon (IFN) to promote immunosuppressive immune cells polarization. Moreover, T_reg_ secreted the TGF-β and IL-35 to enhance the immunosuppression of myeloid-derived suppressor cells (MDSC) [26] (Fig. 1a). Furthermore, T_reg_ showed a more robust function of secreting inhibitory factors along with tumor development and significant increases in the proportion of tumor tissue [27]. Therefore, T_reg_ downregulates the role of effector immune cells and promotes tumor cell proliferation in TIME.

#### Immunosuppressive TAMs

Research has shown that the classification of TAMs based on the expression of CXCL9 and SPP1, rather than the traditional M1 and M2 markers, better correlates with the immune status of tumors [28]. TAMs are a highly plastic cell population that can change their shape and function in response to signals within the tumor immune microenvironment, such as inflammation and metabolic conditions [29]. These changes allow TAMs to promote immune suppression, angiogenesis, and matrix remodeling by secreting various factors like BMP4, CCL18, and CXCL4, which contribute to tumor progression [30] (Fig. 1b). For instance, in various tumors, including lung cancer (LC) and glioma, TAMs play a key role in recruiting and supporting the survival of immunosuppressive cell types while also inhibiting the anti-tumor activity of effector T cells [31–33]. Additionally, tumor cells can influence TAMs to become immunosuppressive by secreting growth factors [34]. In many cancers, monocytes are recruited into the TME and differentiate into immunosuppressive TAMs, primarily regulated by the chemokine ligand (CCL)2/chemokine receptor (CCR)2 and colony-stimulating factor (CSF)-1/CSF-1R signaling pathways. Some clinical trial drugs, such as PF-04136309 and Pexidartenib, have shown promise in reducing the number of immunosuppressive TAMs within tumors [35, 36]. Interestingly, previous studies suggest that TAMs can have an anti-cancer role in the early stages of tumor development, but this gradually shifts towards a pro-cancer phenotype as the tumor progresses [37]. However, the current classification of macrophages does not fully capture their diverse functions, highlighting the need for further refinement. Throughout tumor development, tumor cells influence macrophage polarization through various pathways, with immunosuppressive TAMs shaping the TME to favor tumor cell survival and proliferation.

#### Myeloid-derived suppressor cells

MDSCs promoted the shaping of the immunosuppressive microenvironment and the vascular growth of tumor cells. Immunosuppression is the main feature of MDSCs, and T-cells are their essential targets. MDSCs derived from hematopoietic stem cells and chronic diseases like cancer influenced MDSC differentiation to mature myeloid cells [38, 39]. MDSCs were typically manifested as polymorphonuclear MDSCs (PMN-MDSCs) and monocytic MDSCs (M-MDSCs) in TIME, with phenotypes and functions similar to those of neutrophils and monocytes, respectively [40]. Moreover, MDSCs produced angiogenic and immunosuppressive chemicals that contribute to TIME, secreting IL-10, arginase, and COX2 aiming to restrain anti-tumor cell activity significantly. The VEGF, Bv8, and MMP9 secreted by MDSCs impacted tumor expansion and TIME remodeling [41]. Moreover, CXCL5 and IL-1β secreted by tumor cells correlated significantly to PMN-MDSCs accumulation, and antagonism against IL-1β or CXCR2 retarded tumor growth. The recruitment and enrichment of MDSCs in TIME are facilitated by the Hippo-YAP signaling pathway activation through the CXCL5-CXCR2 axis [42] (Fig. 1c). Interestingly, MDSCs recruitment is stimulated by tumor-derived exosomes through CXCL12-CXCR4, while peripheral PMN-MDSCs correlated to tumor grade, suggesting prognostic significance [43–45]. Furthermore, group 2 innate lymphoid cells in TIME enhanced MDSCs aggregation. In acute promyelocytic leukemia, the induction of innate lymphoid cells significantly increased the proportion of activated M-MDSCs, ultimately promoting immunosuppressive TIME formation. In mouse tumor models, innate lymphoid cells promoted triple-negative breast cancer (TNBC) lung metastasis through the IL-13 axis, whereas IL-13 blockade delayed pro-tumor TIME establishment [46, 47]. Multiple cellular and non-cellular mechanisms with cancer and immune cells promote the immunosuppressive function of MDSC recruitment to TIME. Consequently, MDSCs can modulate tumor proliferation and metastatic potential via a spectrum of mechanisms encompassing both immunosuppressive and non-immunosuppressive modalities. Notably, the non-immunosuppressive modalities include processes such as epithelial-mesenchymal transition, extravasation, dissemination, establishment of pre-metastatic niches, and reactivation of quiescent tumor cells.

### Downregulate recognition of tumor cells recognized by immune cells

Tumor cells altered their protein expression and assisted in immunosuppressive TIME formation to evade immune monitoring. Tumor cell modification included loss or alteration of antigenicity and diminished immunogenicity. The epigenetic information represented by DNA methylation and histone modification was significantly changed in tumor cells. In tumors with high immune cell infiltration, tumor cells can reduce their visibility to the immune system by mechanisms such as loss of heterozygosity in human leukocyte antigen alleles and diminished copy numbers of genes related to antigen presentation. Conversely, in tumors characterized by low immune infiltration, tumor cells actively reduce the number of neoantigens derived from the main group of cancer cells. Tumors with mixed immune infiltration, having areas of both high and low immune activity, present a more complex scenario. In these areas, tumor cells suppress the expression of neoantigens from the dominant clone to avoid immune detection [48]. Additionally, mutations in tumor cell genes can affect the tumor microenvironment and promote immune escape. The continuous activation of particular signaling pathways in tumor cells prevented cell apoptosis and induced chronic inflammatory responses. Alterations in signaling pathways including JAK/STAT, PI3K/AKT, and BRAF-MAPK also supported tumor cells evading recognition by immune cells [49].

Moreover, tumor cells reshape immunosuppressive TIME through various immunoediting methods [50]. A primary source of cellular heterogeneity is the production of tumor neoantigens. Additionally, the function and quantity of some immune cells changed with tumor occurrence and development. Conventional type 1 DCs (cDC1) elicit anti-tumor immunity, thus, tumor cells may limit antitumor immune responses by inhibiting cDC1 recruitment at an early stage [50, 51]. Moreover, deficiencies in human leukocyte antigen (HLA)-like antigen presentation component production and function in cancer cells enhanced immune escape, including genetic and non-genetic reasons for decreased HLA-1 expression and the exhaustion of the antigen processing and presentation component [52]. After the HLA-1 molecular pathway is downregulated, NK cells participate in eliminating tumor cells. This involves the upregulation of the NK cell inhibitory receptor and the shedding of the activating cytotoxic receptor (Fig. 1d). Consequently, the function of NK cells is inhibited through a sequence of mechanisms [50].

In conclusion, tumor cells reshape TIME by endogenous and exogenous factors, eventually achieving immune escape and tumor progression.

## Single-cell landscape of TIME in early-stage cancer

**Fig. 2:**
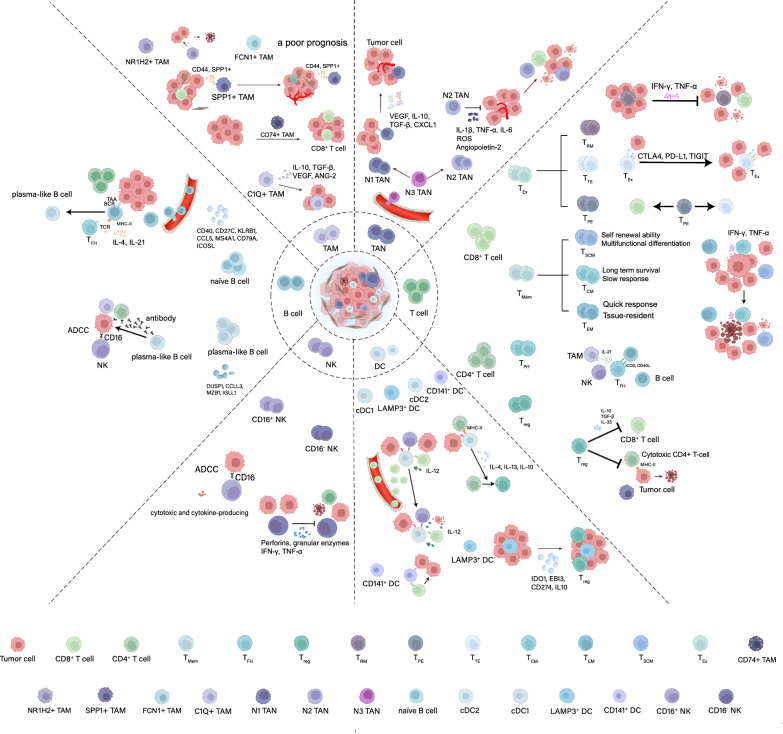
The cellular and molecular landscape of TIME in various tumor progression at single-cell resolution. The major types of immune cells and their subpopulations in TIME include T-cells, B-cells, tumor-associated macrophages, dendritic cells, tumor-associated neutrophils, and natural killer cells. T_Ex_, exhausted T-cell; T_EM_, effector memory T-cell; T_RM_, tissue-resident memory T-Cell; T_h_, help T-cell; T_reg_, regulatory T-cell; TAM, tumor-associated macrophage; TAN, tumor-associated neutrophils; NK, natural killer cell; DC, dendritic cell

Diverse phenotypic subclusters of immune cells have specific functions in tumorigenesis. Single-cell technology has been widely applied to identify and characterize tumor-infiltrating immune cell subsets, distribution, evolutionary lineage, origins, and functions. The scRNA-seq identified and analyzed the momentous TIME alterations during tumorigenesis between tumor tissue and matched adjacent normal tissue or peripheral blood (Fig. 2).

### CD8^+^ T-cells

CD8^+^ T-cells in tumor-infiltrating lymphocytes (TIL) are the most decisive effector cells in anti-tumor response, significantly affecting patient prognosis. The CD8^+^ T-cells can directly recognize and destroy cancer cells and regulate various immune cell development and function.

ScRNA-seq has been widely used to discover new CD8^+^ T-cell subpopulations, analyze cell differentiation trajectories, and explore immune cell interactions. Memory T-cells (T_Mem_) are mainly found in blood and peripheral tissues and can be further divided depending on their persistence and ability to produce effector cell progeny into stem cell memory T-cells (T_SCM_), central memory T-cells (T_CM_) and effector memory T-cells (T_EM_). Exhausted T-cells (T_Ex_) contained terminally exhausted T-cells (T_TE_), progenitor exhausted T-cells (T_PE_), and tissue-resident memory T-cells (T_RM_). The analysis of differentiation trajectories of CD8^+^ T-cells through monocle demonstrated that T_Ex_ was predominantly present in oncogenesis. This analysis can serve as a reference for actual CD8^+^ T cell differentiation [53]. The CD8^+^ T-cell subcluster with IL-17 overexpression promoted tumor cell growth through the cytokine secretion. This T_Ex_, which may originate from T_RM_, is an alternative to the main depletion pathway of CD8^+^ T-cells [54].

From the single-cell perspective of diverse tumors, there were various subpopulations and functional states of CD8^+^ T-cells in TIME, breast [55–57], lung [58], colon [59], liver [15], oral cavity [60], kidney [61], stomach [62], esophagus [63], ovary [64], and nasopharynx [65], including sample sources, major CD8^+^ T-cells subgroups, and main findings of resent studies (Table 1). Single-cell technology compensated for underestimating T-cell subpopulations by traditional classification methods. Compared to adjacent normal tissues, non-functional T-cells were generally enriched in tumor tissues while the number of effector T-cell subsets was reduced. However, the characteristics of CD8^+^ T-cells from different anatomical tissues exhibited variations. Throughout tumor formation, TIME simultaneously included CD8^+^ T-cell subgroups with definite features, and the proportion of CD8^+^ T-cell subpopulations in tumors had significant cancer heterogeneity and patient heterogeneity. For example, two sets of CD8^+^ T_EM_ in gastrointestinal stromal tumors had the highest clonal expansion and cytotoxicity but were also the most exhausted among all T-cells, which has been absent in other tumors [66]. T_Ex_ in TIME overexpressed more immunosuppressive checkpoints, PD-1, CTLA4, and TIGIT, than normal tissue. The variability in expression levels among different subsets was inconsistent, potentially accounting for the significant individual differences observed in the response to immunotherapy [67]. Importantly, scRNA-seq has proved that the progression-free survival of patients with many GZMK + T_EM_ and MKi67 + proliferative subpopulations was relatively high [61, 68, 69]. Briefly, the distribution and function of CD8^+^ T-cells in TIME exhibit significant heterogeneity, with non-functional CD8^+^ T-cells mainly present in tumor tissue and highly expressing immunosuppressive checkpoints. Reprogramming or modulating TIME is a viable therapy to reactivate CD8^+^ T-cell antitumor immune response. ScRNA-seq offers an unparalleled opportunity to understand the heterogeneity, functional states, and interplay of tumor-infiltrating CD8^+^ T cells. This comprehensive analysis can provide profound insights into their contribution to the anti-tumor immune response and explain the varied patient responses to immunotherapy. However, the specific mechanisms of CD8^+^ T-cell exhaustion and potential countermeasures remain unclear. A key direction for scRNA-seq research is a future comprehensive analysis of CD8^+^ T cells, including mechanisms of exhaustion. Additionally, most current studies focus on CD8^+^ T cells, highlighting the need for more in-depth investigation of other immune cells.

**Table 1:** T-cell subsets in TIME characterized by scRNA-seq

Tumor type	Sample	T-cell sorted	Key findings	Refs.
Colorectal cancer Ovarian cancer	Adjacent tissue and tumor Omental metastatic site	CD8^+^ CTL and T_Ex_ cells(XCL1/2) T_RM_ and GNLY^+^CD4^+^ T-cell	XCL1^+^ T-cell clusters are associated with tumor mutational burden high status	[73] [117]
Hepatocellular carcinoma	Tumor and adjacent tissues, and peripheral blood	T_EM_	High T-cell infiltration suggests an anti-tumor response	[68]
Non-small cell lung cancer	Tumor and adjacent tissues, and peripheral blood	T_Ex_	This group of T-cells is clonally expanded, with high migration potentials and state transition possibility	[155]
Breast cancer	Tumor	T_Ex_	T_Ex_ promotes intra-tumoral T-cell exhaustion	[156]
Nasopharyngeal cancer	Tumor and adjacent tissues	CD8^+^ T-cell((LAG-3, TIGIT, PDCD-1 and HAVCR2)	PD-L1^+^ exhausted T-cells are abundant in tumors	[65]
Hodgkin lymphoma	Tumor and reactive lymph nodes	T_reg_-like immunosuppressive subset of LAG3^+^ T-cells	These cell populations may become new therapeutic targets	[157]
Clear cell renal cell carcinoma	Tumor and peripheral blood	MKI^+^ CD8^+^ T-cell	The group of LAG3^+^ T-cells contribute to the immune escape phenotype	[61]
Oral squamous cell carcinoma	Tumor and adjacent tissues	T_Ex_	MKI67^+^proliferative subpopulation is a potential culprit for tumor progression	[60]
Cholangiocarcinoma	Tumor	T_reg_ cells and partial GZMB^+^ CD8^+^ T-cells (LAIR2)	Enrichment of related T-cells is associated with tumor immunosuppression	[158]
Melanoma	Tumor and adjacent tissues, and peripheral blood	T_Ex_	LAIR2 is a potential marker for exhaustive T-cell populations, correlating with the worse survival of patients	[155]
Gastric cancer	Tumor and adjacent tissues	T_EM_, T_RM_, native CD4^+^ T-cells, T_h_, T_fh_, and T_reg_	T_Ex_ promotes intra- tumoral T-cell exhaustion	[62]
Esophageal squamous cell carcinoma	Tumor and adjacent tissues	T_ex_(CCL5, STMN1, and TIGIT), T_reg_(IL1R2)	T_reg_ significantly enriches an immunosuppressive microenvironment T_ex_ and T_reg_ lead to an immunosuppressive microenvironment	[63]

### CD4^+^ T-cells

The tumor-infiltration of CD4^+^ T-cells in TIME is complicated. CD4^+^ T-cell subpopulations can also enhance or suppress the anti-tumor effect of immune cells by coordinating multiple immune responses [70].

The clone expansion of most CD4^+^ T-cells was generally low. However, tumor-infiltration T_reg_ clone expansion was significantly higher. ScRNA-seq has demonstrated that T_reg_ and follicular helper T-cells (T_fh_) in CD4^+^ T-cells played an immunosuppressive role, and T_reg_ with overexpression of immunosuppressive genes was associated with poor survival in patients [62, 71]. Regarding the genealogical differentiation of CD4^+^ T-cells, the scRNA-seq data indicated that naive CD4^+^ T-cells transformed into T_reg_ in primary liver cancer TIME, which may be correlated with macrophages. TAM and naive CD4^+^ T-cells communicated in the IL16 signaling pathway through autocrine and paracrine mechanisms. Compared to adjacent tissues, immunosuppressive CD4^+^ T-cell subpopulations, including T_reg_ were more enriched in tumor tissue [66]. Interactions between CD4^+^ T-cell subclusters and with other immune cells influenced tumorigenesis. David et al. [72] performed scRNA-seq to illustrate that T_reg_ and cytotoxic CD4^+^ T-cells were the critical immune cell clusters influencing the immune response and targeted treatment effect of bladder cancer. Cytotoxic CD4^+^ T-cells relied on MHCII antigens to destroy tumors and were inhibited by autologous T_reg_. However, the surface of tumor cells lacked the expression of MHCII antigens, and whether T_reg_ blocked cytotoxic cells function still needed to be verified. Besides, the analysis of CRC by scRNA-seq discovered that the proportion and crosstalk between T helper cells 1 (T_h1_), T_h17_, and T_reg_ may determine the TIME state. Moreover, the activated T_h_ in TIME can accurately optimize cDC1, thereby promoting the anti-tumor effect of cDC1 based on CTL. This result was validated in mouse experiments, suggesting a potential correlation between CD4^+^ T-cells and other immune cells [70, 71, 73]. Immunosuppressive CD4^+^ T-cell subpopulations are more abundant in TIME, and the crosstalk between CD4^+^ T-cell subclusters and other immune cells affects tumorigenesis.

Thus, the balance between pro-tumor and anti-tumor activities of CD4^+^ T cells plays a critical role in shaping the immune landscape of the TME. Indeed, scRNA-seq is currently being used to explore the diverse functional phenotypes of CD4^+^ T cells. While scRNA-seq offers detailed insights at the cellular level, its ability to detect rare CD4^+^ T-cell subsets with low-abundance transcripts is limited. This could potentially lead to missing important biological information. Looking ahead, developing more efficient and cost-effective technologies is crucial for in-depth studies of CD4^+^ T cells.

### B-cells

Although the proportion is relatively small, B-cells are also a primary component of TIME. Their RNA transcriptome and immunoglobulin secretion properties suggest their distinct anti-tumor immune function.

Traditionally, B-cells were divided into naive B-cells, memory B-cells, and antibody-secreting cells. Recently, scRNA-seq has further subdivided the groups and functions of B-cells and demonstrated the interaction between B-cells and other immune cells. Table 2 shows how the infiltration of B cell subpopulations in the tumor immune microenvironment from various tumor samples can promote or inhibit tumor progression, either directly affecting tumor cells or indirectly interacting with other immune cells at the single-cell level. Unswitched and switched memory B-cells were two sub-clusters of memory B-cells. Unswitched memory B-cells are precursors of long-lived memory B-cells and plasma besides promoting CD4^+^ T-cell differentiation. Meanwhile, switched memory B-cells are precursors of anti-inflammatory specific memory cells and plasma besides promoting antigen presentation [74, 75]. Some B-cell subsets were associated with a positive immune response and a favorable prognosis in patients with nasopharyngeal carcinoma (NPC) [76], breast cancer (BC) [77], and non-small cell lung cancer (NSCLC) [78]. Moreover, scRNA-seq has proven that B-cells in TNBC were more characteristic than memory B-cells. Meanwhile, naive B-cells were enriched in peripheral blood, mutated into memory B-cells under the induction of immune editing and recruited into tumor tissue [77]. ScRNA-seq offers a stronger explanation for the high diversity of B cell subsets observed in various tumors.

**Table 2:** B-cells subclusters in TIME-based on single-cell studies

Tumor type	Sample	B-cell subsets	Key findings	Ref
Breast cancer	Tumor	ICOSL^+^ B-cell (CR2, CD20, CD38/27, and IgA-IgD-)	B-cells elicit anti-tumor T-cell immunity by ICOSL	[159]
Colorectal cancer	Adjacent tissue precancerous tissue and tumor	B-cell (CD40/27, KLRB1, and CCL5), plasma cell(MZB1, DUSP1, and CCL3)	CD40^+^/27^+^ B-cells decrease antigen presentation and antitumor immunity	[73]
Melanoma	Tumor and peripheral blood	B-cells (MZB1, JCHAIN, and IGLL5)	B-cells can promote tumor immunotherapy response	[75]
Non-small cell lung cancer	Tumor	Naïve-like B-cells, plasma-like B-cells	Two specific populations of B-cells regulate the growth of lung tumor cells	[78]
Hepatocellular carcinoma	Tumor and adjacent tissue and lymph nodes	B-cells (MS4A1 and CD79A), plasma cells (IGLL1 and MZB1)	The infiltration levels of B-cells and plasma cells are low in liver cancer	[74]

Furthermore, scRNA-seq can identify B-cell distribution, phenotype, and crosstalk in different tissues. Some B-cell phenotypes were enriched in tumor tissue compare to adjacent regions, with its most significant increase in the germinal center, as revealed by LC and melanoma studies [79, 80]. The total B-cell abundance in NSCLC was less enriched than adjacent tissue, possibly due to inflammatory cell infiltration [78]. Additionally, the expression characteristics of B-cell subpopulations differ between tissues. For instance, B-cell distribution varied between cancers and subtypes. B-cell infiltration in colon cancer and skin melanoma was significantly higher than in esophageal squamous cell carcinoma (ESCC) and hepatocellular carcinoma (HCC) [63]. In the same type of tumor, B-cell proportions differed in distinct human papillomavirus (HPV) infection states. The TIME of HPV-negative head and neck squamous cell carcinoma (HNSCC) had fewer tumor-infiltrating B-cells [81]. The positive role of tumor-infiltrating B-cell subpopulations in oncogenesis, NSCLC and CRC, changed with tumor progression, as confirmed by scRNA-seq studies. This finding further indicates that B-cell subset heterogeneity is vital in immunotherapy and cannot be ignored [78, 82]. Recently, B-cells mainly contributed to the function of T-cells and interacted more with T-cells, as indicated by scRNA-seq studies. Additionally, B-cell maturation depends on the subpopulation of CD4^+^ T-cells (T_fh_) [83]. The crosstalk between the follicular B-cells and T-cells inhibits various tumor cell growth [77]. Interestingly, a cluster of immunosuppressive B-cell subpopulations, known as regulatory B-cells, secreted immunosuppressive factors and interacted with other immunosuppressive cells to produce pro-tumor functions [84].

Research on scRNA-seq of tumor-infiltrating B cells faces limitations. Our understanding of their differentiation pathways and functional phenotypes from a single-cell view remains incomplete. The role of B cells in tumors is unclear and requires more study, especially considering the diverse B cell subgroups across tissues. Additionally, future research should investigate the link between B cell subsets and immunotherapy outcomes. While tumor-infiltrating B cells' transcriptome suggests anti-tumor potential, their specific roles, interaction networks, and impact on immunotherapy demand further exploration.

### NK cells

NK cells are involved to a great extent in immune monitoring and cancer immunity. Their existence and activity are related to the TIME status that affects tumorigenesis. Single-cell sequencing revealed the biological characteristics of tumor-infiltrating NK cell subpopulations. Moreover, gene regulation network analysis suggested that the transcription factors that may regulate tumorigenesis in NK cells were overexpressed, whereas their down-regulation would affect cytotoxic effector expressions including Granzyme B and Perforin-1 [85]. The scRNA-seq was performed to analyze NK cells in HCC, revealing that NK cell activity was higher in paracancerous tissue than in cancer tissues [86]. A low percentage of NK cells in TIME promoted disease progression in gastric cancer [87]. However, in solid tumors, NK cells were usually dysfunctional. According to paired single-cell analysis, the number of cytolytic NK cells in early lung adenocarcinoma (LUAD) was significantly lower than in normal lung tissue, and their anti-tumor function was impaired [88]. To illustrate the potential mechanisms, Ni et al. [89] performed scRNA-seq to define the cell landscape and crosstalk network of mouse tumor-infiltrating NK cells. The results revealed that low hypoxia inducible factor (HIF) 1A expression was associated with high IFNG expression in NK cells. Moreover, the results indicated that NK-IL18-IFNG signals enrichment in solid tumors was associated with increased overall patient survival. Taken together, targeting specific factors restores the anti-tumor properties of NK cells. By uncovering the detailed interactions and functional states of NK cells, scRNA-seq paves the way for novel therapeutic strategies. This could revolutionize cancer prevention and treatment through the development of more targeted and effective NK cell-based therapies. However, scRNA-seq exhibits limitations in analyzing the full functional diversity of NK cell subpopulations and capturing the dynamic interactions between NK cells and other cells within the TME.

### Tumor-associated macrophages

The functions and transcriptional profiles of traditional M1- and M2-TAM exhibited distinct anti- and pro-inflammatory characteristics, respectively. The M1-TAMs directly release cytotoxic factors including nitric oxide (NO) to destroy tumor cells. Meanwhile, tumor cells induce TAM polarization towards M2 via various mechanisms to exert immunosuppressive functions [90]. Moreover, TAMs stimulate tumor vascular growth and immunosuppressive TIME formation, promoting cancer development [91, 92]. The progression of tumors is closely related to the proportion of TAM subpopulations.

Importantly, scRNA-seq has proclaimed that TAMs showed higher phenotypic plasticity and heterogeneity. Consequently, the conventional M1/M2 classification fails to comprehensively elucidate TAM characteristics in patients with various tumors, including colon cancer [93]. TAMs frequently co-express classic M1/M2 genes in glioma [93], liver cancer [94], CRC [95], and renal cancer patients [96]. Additionally, scRNA-seq studies have identified and characterized novel TAM subpopulations in different tumor tissues, revealing high heterogeneity of TAM phenotype. ScRNA-seq identified various subgroups of TAMs beyond the traditional M1/M2 classification, including C1Q^+^ TAMs, SPP1^+^ TAMs, and FCN1^+^ TAMs (Table 3). Specifically, these macrophages with high expression of C1Qs have enhanced phagocytosis, which can eliminate apoptotic cells to prevent autoimmunity. The recruitment and regulation of T-cell subpopulations by C1Q^+^ TAMs affected the functional status of TIME [94, 95]. Another subcluster enriched in tumor tissue is SPP1^+^ TAMs, which regulate the functional state of TIME and tumor angiogenesis, thereby promoting tumor progression. The scRNA-seq analysis revealed that SPP1^+^ TAMs were mainly enriched in CRC and associated with immune checkpoint suppression [95]. Conversely, FCN1^+^ TAM infiltration of originating from monocytes was relatively higher in tissues adjacent to liver cancer [94], LC [97], and CRC [95]. Considering the diverse cellular transformation pathways and heterogeneity across different cancers, a comprehensive understanding of nutrient acquisition and metabolic pathways in TAMs, along with their metabolic interplay within the TME, is crucial for developing cancer treatment strategies targeting TAM mechanisms. Consequently, delineating TAM subgroups using scRNA-seq holds promise to expedite progress in patient stratification and immunotherapeutic approaches. Therefore, identifying TAM subgroups by scRNA-seq may accelerate the development of patient clinical stratification and immunotherapy.

**Table 3:** TAM and DC subpopulations in TIME identified by scRNA-seq

Tumor type	Sample	Subsets	Key findings	Ref
Colorectal cancer	Tumor and adjacent tissue precancerous tissue	TAM (SPP1^+^, CXCL5^+^, C1QC^+^, and CD55^+^)	These subgroups have both pro-inflammatory and anti-inflammatory effects	[73]
Clear Cell Renal Carcinoma	Tumor and adjacent tissues	TAM (C1Q^+^TREM2^+^APOE^+^)	The group of TAM leads to tumor recurrence	[160]
Ovarian cancer	Tumor	TAM(NR1H2, IRF8, and CD274)	This macrophage cluster has anti-tumor effects	[117]
Hepatocellular carcinoma	Tumor and adjacent tissues and ascites and peripheral blood, and lymph nodes	CD74^+^ TAM	The CD74^+^TAM increases the infiltration of CD8^+^ CTL and enhances effector functions in HCC	[161]
Prostate Cancer	Tumor and adjacent tissues	TAM (SLC40A1, PLAC8, and FCN1)	The group of TAMs has a poor prognosis in patients	[162]
Hepatocellular carcinoma	Tumor and adjacent tissues and ascites and peripheral blood, and lymph nodes	LAMP3^+^ DC	The group of DCs has the potential to migrate from tumors to LNs	[94]
Nasopharyngeal carcinoma	Tumor	CLEC9A^+^ DC	This cell population is expected to be a new therapeutic target	[85]

### Tumor-associated neutrophils

Tumor-associated neutrophils (TANs) can either promote or inhibit tumor growth depending on their polarization state: anti-tumor TAN-1 and pro-tumor TAN-2. TANs drive angiogenesis and extracellular matrix remodeling to promote tumor inflammatory response. Additionally, TANs directly destroy tumor cells and participate anti-tumor TIME formation.

Xue et al. [98] 2022 used scRNA-seq to comprehensively analyze the TAN cellular and molecular landscape in human and mouse primary liver cancer. The results revealed that neutrophils exhibited heterogeneity in different TIME subtypes. TANs primarily express immunosuppressive features, with CCL4^+^ TANs recruiting macrophages and PD-L1^+^ TANs damaging T-cell cytotoxicity. The results further explored the biological features of TANs, indicating their involvement in primary LC occurrence. To analyze the specific neutrophil subpopulations promoting tumors, Wang et al. [17] 2023 performed scRNA-seq to characterize the heterogeneity and mechanisms of pancreatic ductal adenocarcinoma (PDAC). The results showed four TAN subpopulations with different functional characteristics, containing TAN-1/TAN-2/TAN-3/TAN-4. Beyond TAN-1 and TAN-2, scRNA-seq has identified and characterized other novel TAN subgroups at the single-cell level, helping to explain the heterogeneity of the tumor microenvironment. TAN-3 was a subgroup that had just migrated from peripheral tissues to TIME. Importantly, TAN-1 was a pre-tumor subpopulation characterized by an overactive glycolysis pathway. Hypoxia and BHLHE40 were critical regulators of TAN differentiation to the TAN-1 phenotype. ScRNA-seq technology sheds light on the intricate heterogeneity of TANs by elucidating their functional states and interactions with tumor cells within the tumor microenvironment. This approach provides valuable insights; however, research employing scRNA-seq to investigate TANs remains relatively limited. While existing studies suggest a significant role for TANs in tumor development, these findings are preliminary. Further investigation with larger sample sizes is necessary to validate the functional significance of TAN subpopulations identified through scRNA-seq analysis.

### Dendritic cells

Tumor-infiltrating dendritic cells (DCs) are mainly divided into four subgroups: plasmacytoid DCs (pDC), cDC1/2, and migratory DCs (migDC) [99]. Some novel DC subpopulations revealed by scRNA-seq were strongly associated with immune response. Table 3 highlights novel DC subpopulations revealed by scRNA-seq that are strongly associated with immune response, such as LAMP3^+^ DCs and CLEC9A^+^ DCs. Notably, CD141^+^ DCs exhibited exhaustion characteristics in scRNA-seq data of early LUAD. Using scRNA-seq, pDCs were found to significantly improve the prognosis of NPC patients besides identifying a unique subtype of DCs. However, precise mechanism through which this novel subgroup hinders the functioning of other immune cells remains uncertain [85]. Moreover, the scRNA-seq characterized the paramount new subgroups, LAMP3^+^ DCs, and analyzed their origin, infiltrating distribution, functional characteristics, and crosstalk in detail. This finding proves the possible mechanism of LAMP3^+^ DCs in affecting T-cell subpopulation infiltration and immunotherapy response, DCs induced tumor-specific cellular and humoral immune responses, resulting in a reduction in tumor volume and the establishment of immunological memory [63, 94]. Furthermore, the LAMP3^+^ DCs subgroup accelerated immunosuppressive TIME formation in bladder cancer by recruiting T_reg_ [100]. The study showed an opposing trend of T_regs_ in autoimmune diseases compared to cancer, suggesting fundamentally different treatment approaches are needed for these two conditions [101]. Using scRNA-seq has provided insights into the role of DCs in regulating immune homeostasis in ESCC, as traditional DCs inhibit CD8^+^ T-cell activation [102]. Leveraging the potent antigen-presenting capacity of LAMP3^+^ DC provides a pathway for developing cancer vaccines that target specific tumor antigens. Activation of T-cell responses against these tumor-specific antigens has the potential to significantly improve the therapeutic effectiveness against tumors. The clinical efficacy of DCs as tumor vaccines was inadequate, and more scRNA-seq studies are needed to find potential immune targets.

Collectively, scRNA-seq comprehensively identifies and characterizes unsuspected immune cell subsets and functional mechanisms in early cancer TIME, providing more insights into tumorigenesis biology. While scRNA-seq identifies DC characteristics and functions, validation through in vitro and in vivo experiments is necessary. However, these validations are limited by model selection and experimental conditions.

In the context of tumorigenesis, scRNA-seq has been instrumental in revealing the complexity and heterogeneity of immune cells, offering insights into their roles and interactions. However, its application is limited by challenges such as capturing the full spectrum of dynamic immune responses over time and the difficulty in comprehensively understanding the interactions between immune cells and the tumor microenvironment due to the technique's snapshot nature.

## Single-cell landscape of TIME in advanced or metastatic solid tumor

TIME is involved in creating a favorable "soil" for circulating tumor cells. Therefore, tumor cells and immune cells interact upon metastasis. The scRNA-seq can be applied to identify and characterize immune cells or their metastasis subsets, thereby discovering new targets and improving clinical efficacy in late tumors [103].

### TIME of advanced or metastatic cancer

The metastasis of advanced tumors to various organs is a common occurrence, and as such, this section will provide a separate summary based on cancer types.

#### Cervical cancer

Our team conducted scRNA-seq to profile the cellular landscape of different pathological subtypes of cervical cancer and cellular and molecular variations in tumors treated with various therapies [104–107]. Importantly, we collected 13 human cervical tissue samples at different clinical stages for scRNA-seq to observe the characteristic variations of specific cell subpopulations from normal epithelium to tumor metastasis. Subsequently, we analyzed the cell abundance and functional alterations of cervical squamous cell carcinoma at various stages. The results revealed that the abundance of some immune cell subclusters was significantly correlated to tumor progression. Specifically, CCL20^+^ and APOE^+^ macrophages with high anti-inflammatory characteristics were enriched in the advanced cervical squamous cell carcinoma. Meanwhile, the abundance of CD16^+^ NK cells was relatively low and negatively correlated to survival. The decrease of CLEC9A^+^ DC with tumor progression indicated a better prognosis. In addition, T_h17_ and TNFRSF9^high^ T_reg_ were enriched and highly expressed CCL20 in advanced cervical squamous cell carcinoma. Moreover, this gene expression level was associated with poor prognosis [108]. We provided paramount insights into key cell populations and molecular variations in tumor occurrence and development.

#### Breast cancer

As previously described, most BC metastases have decreased immune cell expression, whereas macrophage characteristics were preserved [109]. The scRNA-seq enabled further analysis of immune cell subclusters and underlying molecular mechanisms. In BC metastasis, immune cell number decreased, whereas the immunosuppressive cell proportion increased significantly [110]. Moreover, fatty acid metabolism polarized immune cells to pro-tumor phenotype for energy supply, suggesting that cutting off this pathway may delay BC progression [111]. Devon et al. [112] used scRNA-seq to analyze the TNBC metastasis model and confirmed that metastatic tumor cells preferentially utilized oxidative phosphorylation (OXPHOS) to provide energy compared to primary BC. Therefore, BC metastases are more inert in TIME at the single-cell resolution. The inhibition of tumor metastasis can be achieved by targeting specific immune alterations and metabolic pathways. ScRNA-seq analyzes the transcriptome and heterogeneity of the immune microenvironment in advanced BC at the single-cell level, revealing mechanisms of metastasis and drug resistance.

#### Osteosarcoma

The scRNA-seq also identified the immunosuppressive status of osteosarcoma metastases, a lower proportion of CD4^+^/8^+^ T cells. Interestingly, FABP4^+^ TAM expressing pro-inflammatory features was identified in osteosarcoma lung metastases. This paradoxical conclusion illustrated that tissue-resident macrophages may also be involved in osteosarcoma metastasis. Furthermore, CCR7^+^ DC, a subpopulation of DC, was associated with osteosarcoma lung metastasis [113]. Liu et al. [114] further studied the distinct DC population at the single-cell level, demonstrating that regulatory DC cluster assisted tumor cells in evading immunity by recruiting T_reg_. The results indicated that the decreased tumor immunogenicity and expression of the novel signal CD24 were potential mechanisms of immune evasion in advanced osteosarcoma. Additionally, cDC2 has demonstrated promising therapeutic outcomes in cancer treatment by enhancing CD4^+^ T-cell immune responses [115]. ScRNA-seq unveiled the multicellular ecosystem in primary and metastatic osteosarcoma tissues, providing insights into its development, progression, and potential cell therapy strategies.

#### Ovarian cancer

Advanced ovarian cancer (OC) is prone to widely disseminated metastases, including pelvic and abdominal planting spread [116]. However, the function of specific immune cell subclusters that may influence OC progression was unknown. Therefore, Olalekan performed scRNA-seq to divide metastatic OC into high- and low- T-cell infiltration groups, exhibiting various immune cell cluster infiltration in metastatic OC. Immune cells are mainly distributed around adipocytes, possibly due to energy acquisition. The high-T-cell infiltration group included T_RM_ with high TOX gene expression, CD4^+^ T-cells with granulysin expression, plasma cells, and NR1H2^+^ IRF8^+^ and CD274^+^ macrophage subsets. These results suggest that the high- T-cell infiltration group exhibits an anti-tumor response [117]. Besides, Xu et al. [118] used scRNA-seq and found that special subclusters were relatively reduced in advanced high-grade serous OC, comprising M1-TAM, T_RM_, and T_Ex_. Notably, TIGIT blockade therapy effectively inhibited OC metastasis in mouse models, consistent with osteosarcoma [113]. Therefore, these results showed changes in the number of some immune cells in advanced OC. Consequently, some novel immune checkpoints may become targets for metastatic tumors. Single-cell immune characteristics obtained by scRNA-seq enable patient classification for guiding treatment and prognosis in OS.

#### Lung cancer

The TIME anti-tumor properties of LC metastasis were diminished, resulting in low efficacy of immunotherapy in some patients. The scRNA-seq thoroughly characterized the immune landscape of LC in different stages. This investigation provided that LC heterogeneity was primarily caused by macrophages and lymphocytes [119, 120]. Many T-cell-based immunotherapies have been developed for LC, hence this section mainly focused on immune cells other than T- cells.

Kim et al. [79] employed scRNA-seq to reveal the cellular and molecular characteristics of the TIME from a precancerous lesion to late LUAD. The results indicated that TAM and DC gradually replaced normal myeloid cells. Moreover, T_Ex_ and mononuclear macrophages (mo-Mac) increased significantly in metastases or late tumors, which may promote tumor metastasis. Eventually, they confirmed the molecular interactions between immune cells and cancer cells during LUAD development. Mo-Mac and malignant cells promoted the growth of each other via different pathways. Furthermore, mo-Mac regulated T_Ex_ to balance immune activation and inhibition. Consequently, targeting mo-Mac represented a novel therapeutic avenue. Based on the scRNA-seq, Chen et al. [78] confirmed naïve-like and plasma-like B-cells and their paradoxical functions developing NSCLC. The two major subclusters were also validated by flow cytometry and immunohistochemistry. The findings suggested that in terminal NSCLC, naive-like B-cells can inhibit LC tumor cell growth while plasma-like B-cells assist LC cell growth. Another scRNA-seq study unveiled immunosuppressive cells in the cerebrospinal fluid of LC brain metastases [111]. The scRNA-seq of macrophages isolated from mouse model lung metastases revealed extensive heterogeneity of TAM, with newly identified subpopulations involved in lipid metabolism, extracellular matrix remodeling, and immune suppression [93]. Thus, these immune cell subpopulations are potential therapeutic targets for patients with poor immunotherapy efficacy. ScRNA-seq has contributed to novel treatment plans, offering hope for patients with limited treatment options.

#### Pancreatic ductal adenocarcinoma

Immune cells are crucial in the development of PDAC. An often-neglected pathway in PDAC is perineural infiltration, which warrants attention due to its significance. The importance of perineural infiltration and the role of TAM in PDAC implied that immune cells could be potential therapeutic targets for blocking perineural infiltration [121]. Liu et al. [122] conducted the scRNA-seq on PDAC samples and drew the TIME outlook of primary and metastatic tumors. The results proved that tumor cells escaped NK-mediated immune monitoring with the assistance of platelet-derived RGS18 and immune checkpoint HLA-E: CD94-NKG2A. Moreover, the metastatic site TIME cell landscape appeared more concise at the single-cell level. By constructing a TIME heterogeneity map of PDAC, scRNA-seq identified immune cell subpopulations that differentiate metastatic from non-metastatic samples, aiding patient prognosis. Lin et al. [64] analyzed ten primary tumors and six metastatic tumors of PDAC using scRNA-seq. The results showed that metastatic tumor TIME was more inert, with TILs displaying similar functionality. Conversely, TAMs formed distinct clusters, suggesting that these two macrophage phenotypes serve different roles. Overall, immune cells identified by scRNA-seq contribute to defining of PDAC subtypes and correlate with patient prognosis.

#### Colorectal cancer

The liver is a common metastatic site in CRC patients. Wu et al. [123] 2022 examined scRNA-seq to analyze CRC liver metastasis. The results indicated that immunosuppressive cells were overrepresented in CRC liver metastasis and immune cells influenced tumor progression in space. Additionally, they developed single-cell metabolism (scMetabolism), a method for calculating metabolism at the single-cell level. The results revealed that the metabolism of MRC1 CCL18^+^ M2-like macrophages was the highest, suggesting that inhibiting the metabolic pathway may also slow tumor progression. Moreover, Liu et al. [18] developed the PhenoAligner algorithm by scRNA-seq to quantify the effects of niche and malignancy on immune cell phenotypes. They also defined immune taxa whose phenotypes were significantly influenced by tumor cell characteristics and organ microenvironment. The results revealed that immune cell infiltration influenced pathological states and the organs in which they resided. Additionally, key immune cell subpopulations were found to be associated with tumor progression or metastasis, including the SPP1^+^ TAM enriched in CRC liver metastasis. ScRNA-seq has elucidated the roles of immune cell subsets in CRC metastasis, providing potential diagnostic markers and therapeutic targets. Furthermore, scRNA-seq analyzed mouse models of liver metastasis to explore metastatic tumor formation. They detected that TAM induced T-cell dysfunction through the Fas-Fasl pathway and identified the potential mechanisms of different transfer sites TIME [124]. Thus, metabolic and signaling pathways can also block tumor metastasis.

#### Melanoma

Early melanoma can be entirely eradicated by surgical resection, but advanced melanoma is ineffective for systemic treatment [125]. Fischer et al. [126] performed scRNA-seq to comprehensively analyze the TIME landscape of paired melanoma brain metastasis (MBM) and extracranial metastasis (ECM). The overall level of immune suppression in MBM was higher than in ECM, specifically manifested in a significant reduction in CD3^+^/8^+^ T-cells, monocytes, and DCs, while a relative increase in TANs. Furthermore, inhibiting the OXPHOS pathway can prevent melanoma from metastasizing to the brain. Compared to MBM and skin metastases, the immunosuppressive TIME of leptomeningeal metastases was mainly reflected in the dysfunction of CD4^+^/8^+^ T-cells. Leptomeningeal metastases TIME contained the highest proportion of exhausted and apoptotic CD4^+^ T-cells, and the number of CD8^+^ T-cells was relatively small. Among the three types of metastatic tumors, leptomeningeal metastases had the lowest B-cell content [127]. Another scRNA-seq study of MBM demonstrated that the immune response of patients was positively correlated to myelocyte heterogeneity and the functional status and quantity of T-cells [128]. These results identify the unique characteristics of melanoma metastasis in different regions. ScRNA-seq identified novel resistance pathways in multiple myeloma, including hypoxia tolerance and mitochondrial respiration. Notably, it validated peptidylproline isomerase A as a promising therapeutic target.

#### Head and neck squamous cell carcinoma

Lymph nodes were also a common site of metastasis in HNSCC. Puram et al. [129] 2017 analyzed five pairs of primary and metastatic HNSCC by scRNA-seq. The number and population of immune cells in their TIME were generally the same, but B and plasma cells were enriched in metastasis. Quah et al. [130] 2023 further resolved the possible immune evasion mechanisms of early HNSCC metastasis using scRNA-seq. Interestingly, plasma cells and DC also accounted for several advantages in lymphatic metastasis. Importantly, two proteins (AXL and AURK) identified by researchers blocked metastasis and SOX4-promoted CD8^+^ T-cell exhaustion. Regulating the protein expression can also alter the function of immune cells, thereby achieving the goal of blocking tumor metastasis. Integrating scRNA-seq with current computational tools has identified potential drug targets in early-stage HNSCC metastasis. This approach holds significant promise for specific clinical applications and for addressing critical issues.

#### Gastric cancer

The clinical symptoms of gastric cancer patients with abdominal metastasis are severe. According to the scRNA-seq study by Wang et al. [131], the immune response of peritoneal metastases derived from mixed gastric and colonic-like cells was more effective, possibly due to the enrichment of M1-TAM and B-cells in TIME and active immune-related pathways. Defensins, IL-7, complement cascade, and IL6/JAK/STAT3 signaling pathways were enriched in tumor tissues. Signet ring cell carcinoma of the stomach is a highly malignant tumor in gastric cancer with a high rate of metastasis and recurrence, unique cytological characteristics, and an immune microenvironment [132]. Chen et al. [133] performed immune-targeted scRNA-seq to analyze the TIME of different subtypes of advanced gastric cancer, demonstrating that activating CD4^+^/8^+^ T-cells in the TIME of gastric signet ring cell carcinoma was challenging, and the normal function of B-cells in the tertiary lymphatic structure was also severely impaired. Notably, CXCL13 was the central coordinator of depletion state transition and can predict the response of gastric cancer patients to immune checkpoint blockade. The evolution and diverse phenotypes of immune cell subpopulations in gastric cancer metastasis were analyzed, shedding light on the interaction and coexistence patterns among immune cells within metastatic gastric tumors.

### TIME of organ-specific metastasis

Unlike the above studies, this section reviews the TIME features of diverse cancers metastasizing to the same site, also known as organ-specific metastasis. Previous studies have provided comprehensive immune landscape resources for primary brain tumors and metastatic tumors through scRNA-seq [55, 134]. TIME was disease-specific, with differences in the characteristics of macrophages, neutrophils, and T-cells. By integrating multiple data, transcriptome, Klemm et al. [135] illustrated differences in immune-cell phenotype and function of brain metastases from various extracranial primary tumors. Notably, the neutrophil abundance was the highest in BC brain metastases, while CD4^+^/8^+^ T-cells were abundant in brain metastases from melanoma, which was the possible reason for the definite effect of immunotherapy on melanoma. These results revealed the disease-specific enrichment and multiple functional activations of immune cells. Friebel et al. [134] also suggested that the subgroups and abundance of immune cells, as well as the activation and quantity of functional states, in brain metastases determine the effectiveness of immunotherapy for different tumors. Gonzalez et al. [136] roughly classified the TIME status of brain metastases; the authors applied single-cell transcriptomics to analyze brain metastases from eight cancer types in detail. The results showed that TIME was in an immunosuppressive state with more T-cells and TAM. The result implied that most T-cells and macrophages were in dysfunctional differentiation subgroups. Notably, the eight functional cell programs promoted by TIME describe two functional states of brain metastases: proliferation and inflammation. These specific immunological features obtained with scRNA-seq promote the rationalization of targeted immunotherapy for organ-specific metastatic tumors. Implementing targeted local treatment based on a single-cell atlas may significantly enhance patient survival due to the potential for prolonged persistence of certain metastases in metastatic sites [137].

ScRNA-seq offers insights into cellular heterogeneity and interactions between immune and tumor cells, revealing diverse immune cell subsets and their functions. It has uncovered potential biomarkers and therapeutic targets, paving the way for precision medicine. However, scRNA-seq provides a static picture, limiting our ability to capture the full complexity of the TME over time. Despite these limitations, the detailed exploration of the TME at the single-cell level remains crucial for advancing our understanding of immune evasion, tumor resilience, and improving treatment strategies for advanced or metastatic tumors.

## Novel immunotherapy targets characterized by scRNA-seq

Different immune cell subsets exert their specific functions through interaction networks. ScRNA seq has been widely applied to study intercellular signaling mediated by secretion factors and its role in tumor progression.

### Cytokines and chemokines

Immune-related molecular variations are involved in tumorigenesis and tumor development. Cytokines and chemokines were principal mediators of immune cell interactions, and some of the previous sections have already been introduced. As mentioned previously, cytokines and chemokines play a key role in mediating interactions between immune cells. For instance, IL-10/35 promotes T_reg_ activity, the CCL2/CCR2 axis recruits immunosuppressive TAMs, and the CXCL5-CXCR2 axis is involved in MDSC function. Antagonizing IL-10 can increase the number of CD8^+^ T-cells and macrophages in TIME, which has an independent destroying effect on tumor cells and increases chimeric antigen receptor (CAR) T-cell therapy efficiency [138]. Preclinical studies have shown that disrupting the CCL2-CCR2 signaling pathway could effectively block the recruitment of immunosuppressive TAMs, suggesting a promising target for manipulating the tumor microenvironment. However, a phase II clinical trial enrolling patients with metastatic castration-resistant prostate cancer found that carlumab, a monoclonal antibody targeting CCL2, was well-tolerated but lacked significant anti-tumor activity as a monotherapy [139].

The differences in the expression levels of receptors and their ligands among diverse immune cells, and the correlation between the expression of these specific ligands and receptors, can serve as important markers of intercellular signaling. By determining signal transmission between single cells, scRNA-seq enables researchers to draw a more precise signal transmission network diagram between cells and identify key molecules or molecular pairs that enhance tumor occurrence and progression [140]. ScRNA-seq can also encourage research on clinically paramount cell group signaling and improve the efficiency of individualized treatment. Chen et al. [133] carried out scRNA-seq to demonstrate that the function of recruiting B-cells for advanced gastric signet-ring cell carcinoma was significantly impaired, precisely due to the significant reduction in the number of cytokines and chemokines produced by T-cells in TIME, particularly CXCL13. Additionally, scRNA-seq proclaimed that the CCL5, CXCL14, CXCL16-CXCR6, and CCL20-CCR6 promote immune escape and metastasis of tumor cells [141–144]. Therefore, targeting these molecules can delay tumor progression. However, the crosstalk between TIME is complex and the detailed mechanisms remained unelucidated. While the significance of chemokines and their receptors in tumors is increasingly recognized, scRNA-seq and similar technologies are enhancing our understanding of tumor dynamics and fostering novel therapeutic approaches. However, translating this knowledge into effective treatments remains challenging. Targeting these molecules requires balancing immune system effects and tailoring treatments to patients. Future studies must investigate these molecules' roles in tumorigenesis and progression and devise ways to convert insights into viable treatments.

### Immune checkpoint

The activity of TILs determines the effectiveness of immunotherapy. Inhibitory immune checkpoints accelerate tumor growth by promoting effector T-cells in TILs dysfunction and exhaustion. programmed cell death protein 1(PD-1) and Cytotoxic T lymphocyte-associated antigen-4 (CTLA-4), widely used clinically, are the two most comprehensively studied immune checkpoints. The function and quantity of immune cells are closely related to clinical efficacy [145–147]. Through scRNA-seq analysis in human tumor samples, Lymphocyte-activation gene 3 (LAG3), T-cell Immunoglobulin and Mucin-domain containing-3 (TIM3), and T cell immunoreceptor with Ig and ITIM domains (TIGIT) were upregulated and co-expressed in CD8^+^ T-cells. Importantly, using scRNA-seq to analyze samples of cervical cancer and osteosarcoma revealed that blocking TIGIT therapy significantly inhibited tumor cell expansion. This result validated the bright prospects of combined or single use of anti-TIGIT therapy in advanced tumors [113, 118]. Furthermore, based on scRNA-seq, TIGIT was the primary participant in the recurrence of mantle cell lymphoma receiving CAR-T. Targeted inhibition of TIGIT simultaneously resulted in prolonged progression-free survival in patients. Some checkpoint inhibitors also increased after recurrence, and their functions were worth further exploration [148]. Interestingly, in the single-cell dataset of NPC, both LAG-3, and TIM3 (HAVCR-2) had the highest expression levels of immune checkpoint genes in CD8^+^ T-cells. This pattern is similar in patients, indicating that other immune checkpoints in TIME, including LAG-3 and TIM3, may be potential targets for checkpoint inhibition [65].

Over the past few years, TIM3 and LAG3 have been extensively investigated as immunotherapy targets following PD-1 and CTLA4 in clinical trials. Studies have shown a significant correlation between TIM3 expression and non-responsiveness to anti-PD-1 therapies, suggesting that elevated TIM3 levels may promote immune evasion. This highlights the potential of combination therapies targeting both TIM3 and PD-1 pathways. TSR-022, a humanized anti-TIM3 IgG4 monoclonal antibody for solid malignancies like NSCLC, HCC, and melanoma, has shown dose-dependent clinical activity in a cohort of 202 NSCLC patients resistant to PD-1/PD-L1 antibodies, particularly when administered with a fixed dose of TSR-042, a PD-1 targeting antibody, without encountering any dose-limiting toxicities [149]. Additionally, RO7121661 represents a bispecific antibody targeting both TIM3 and PD-1 simultaneously, designed to further explore this combination strategy. A phase II clinical trial is actively recruiting patients with advanced metastatic solid tumors. Clinical evaluations of other anti-TIM3 monoclonal antibodies, namely SHR-1702, TQB2618, and Surzebiclimab, are also underway to determine their efficacy in treating advanced malignancies [150]. Similarly, RELATIVITY-047 (NCT03470922) is a randomized, double-blind, phase II/III clinical trial that assessed the efficacy and safety of relatlimab (LAG3 antibody) combined with nivolumab (PD-1 antibody) versus nivolumab monotherapy in previously untreated metastatic or unresectable melanoma. The study met its primary endpoint of progression-free survival and exhibited a favorable safety profile [151]. While TIGIT checkpoint inhibitors have been primarily evaluated in LC clinical trials, preliminary findings suggest modest efficacy as monotherapy [152]. Combining TIGIT inhibitors with PD-1/PD-L1 inhibitors has shown promise in Phase I and II studies, although Phase III trials have not yielded conclusive results yet [153, 154]. The effectiveness of anti-TIGIT drugs in benefiting cancer patients remains under investigation, with some suggesting they might act as sensitizers to enhance the effects of existing treatments.

Consequently, scRNA-seq furnishes potential immunotherapeutic targets by identifying novel immune-related molecules and checkpoints at different stages. However, the anti-tumor function of these targets still needs to be identified by more animal model experiments and clinical trials.

## Conclusion

The scRNA-seq profiles the comprehensive cellular and molecular alterations of TIME in tumorigenesis and metastasis, novel cell subpopulations, cell states, and molecular mechanisms that may promote tumor development. Immune cell subpopulations and molecular pathways play critical roles in tumor progression. Their modulation offers therapeutic potential. ScRNA-seq has yielded significant insights into the tumor immune microenvironment, including immune cell transcriptomics, heterogeneity, evolutionary lineages, and mechanisms of drug resistance and recurrence. It has also identified predictive markers and targets for immunotherapy. Establishing single-cell public databases for some tumors and in-depth analysis of scRNA-seq data are crucial for elucidating the immune characteristics of TIME. Our study emphasizes the complexity of TIME and the necessity of multi-target combination therapy. However, due to technological limitations, we are still unable to showcase the completely personalized characteristics of all tumors. Isolating and capturing single cells can impact immune cell viability and integrity, potentially skewing the assessment of microenvironments. Additionally, despite increased throughput, single-cell technologies still face limitations in sample size, hindering the translation of basic scRNA-seq research into clinical practice. Another limitation is that the single-cell data of immune cells other than CD8 ^+^ T-cells is relatively limited, requiring more horizontal and vertical research. Collectively, our review provides insights into the evolution of diverse tumor immune systems for future research, hoping to improve immune response and identify potential novel targets for immunotherapy.

## Data Availability

Not applicable.

## Abbreviations

TME: Tumor microenvironment
TIME: Tumor immune microenvironment
scRNA-seq: Single-cell RNA sequencing
TAMs: Tumor-associated macrophages
CRC: Colorectal cancer
Treg: Regulatory T-cell
IL-10: Interleukin 10
NK: Natural killer
CTL: Cytotoxic lymphocytes
IFN: Interferon
MDSC: Myeloid-derived suppressor cells
LC: Lung cancer
CCL: Chemokine ligand
CCR: Chemokine receptor
SCLC: Small-cell lung cancer
PMN: Polymorphonuclear
M: Monocytic
TNBC: Triple-negative breast cancer
cDC1: Conventional type 1 DCs
HLA: Human leukocyte antigen
TIL: Tumor-infiltrating lymphocytes
T_Mem_: Memory T-cells
T_SCM_: Stem cell memory T-cells
T_C__M_: Central memory T-cells
T_E__M_: Effector memory T-cells
T_E__x_: Exhausted T-cells
T_TE_: Terminally exhausted T-cells
T_PE_: Progenitor exhausted T-cells
T_RM_: Tissue-resident memory T-cells
Tfh: Follicular helper T-cells
Th1: T helper cells 1
NPC: Nasopharyngeal carcinoma
BC: Breast cancer
NSCLC: Non-small cell lung cancer
ESCC: Esophageal squamous cell carcinoma
HCC: Hepatocellular carcinoma
HPV: Human papillomavirus
HNSCC: Head and neck squamous cell carcinoma
LUAD: Lung adenocarcinoma
HIF: Hypoxia inducible factor
TANs: Tumor-associated neutrophils
PDAC: Pancreatic ductal adenocarcinoma
pDC: Plasmacytoid DCs
migDC: Migratory DCs
OXPHOS: Oxidative phosphorylation
OC: Ovarian cancer
mo-Mac: Mononuclear macrophages
scMetabolism: Single-cell metabolism
MBM: Melanoma brain metastasis
ECM: Extracranial metastasis
CAR: Chimeric antigen receptor
PD-1: Programmed cell death protein 1
CTLA-4: Cytotoxic T lymphocyte-associated antigen-4
LAG3: Lymphocyte-activation gene 3
TIM3: T-cell Immunoglobulin and Mucin-domain containing-3
TIGIT: T cell immunoreceptor with Ig and ITIM domains
